# Role of β-Adrenergic Receptors and Estrogen in Cardiac Repair after Myocardial Infarction: An Overview

**DOI:** 10.3390/ijms22168957

**Published:** 2021-08-19

**Authors:** Paola Matarrese, Sonia Maccari, Rosa Vona, Lucrezia Gambardella, Tonino Stati, Giuseppe Marano

**Affiliations:** Center for Gender Specific Medicine, Istituto Superiore di Sanità, 00161 Rome, Italy; sonia.maccari@iss.it (S.M.); rosa.vona@iss.it (R.V.); lucrezia.gambardella@iss.it (L.G.); tonino.stati@iss.it (T.S.); giuseppe.marano@iss.it (G.M.)

**Keywords:** β-adrenergic receptors, estrogen, inflammation, cardiac repair, gender, preclinical studies

## Abstract

Acute myocardial infarction (MI) is associated with an intense inflammatory response that is critical for cardiac repair but is also involved in the pathogenesis of adverse cardiac remodeling, i.e., the set of size, geometry, and structure changes that represent the structural substrate for the development of post-MI heart failure. Deciphering the pathophysiological mechanisms underlying cardiac repair after MI is, therefore, critical to favorably regulate cardiac wound repair and to prevent development of heart failure. Catecholamines and estrogen play an active role in regulating the inflammatory response in the infarcted area. For example, stress-induced catecholamines alter recruitment and trafficking of leukocytes to the heart. Additionally, estrogen affects rate of cardiac rupture during the acute phase of MI, as well as infarct size and survival in animal models of MI. In this review, we will summarize the role of β-adrenergic receptors and estrogen in cardiac repair after infarction in preclinical studies.

## 1. Introduction

Cardiovascular diseases, including myocardial infarction (MI) and heart failure (HF), are the leading cause of mortality in industrialized countries. Advances in coronary care and cardiac revascularization have reduced early mortality in patients with acute MI. However, the risk of developing HF after MI has remained high. Adverse cardiac remodeling, i.e., the set of size, geometry, and structure changes that occur after the loss of a significant amount of heart muscle, is the structural substrate for the development of post-MI heart failure. A growing body of evidence suggests that post-MI remodeling depends on both the size of the infarct and altered cardiac repair after MI.

MI occurs when the supply of blood to a part of the heart is suddenly blocked, causing necrosis of myocardial tissue. Ischemic events induce damage not only in cardiomyocytes (CMs) but also in vessel cells and cardiac fibroblasts (CFs). The ability of heart tissue to recover after these events is carried out through a complex remodeling process, orchestrated by CFs, inflammatory cells, and CMs [1]. Since the human myocardium has negligible endogenous regenerative capacity, necrotic cell death within the infarcted area triggers an inflammatory response aimed at clearing the infarct from dead cells. This response subsequently activates interstitial cell populations, which have a critical role in reparative mechanisms required to generate a collagen-based scar and to maintain the structural integrity of the infarcted heart. The removal of necrotic tissue occurs due to the activity of immune cells that release enzymes, reactive oxygen species (ROS), growth factors, and cytokines. In particular, infiltrating leukocytes release pro-inflammatory interleukin 1β (IL-1β) and reparative transforming growth factor β (TGF-β1) in the damaged area [2]. Therefore, within certain limits, the inflammatory state that is established in the myocardium following MI promotes homeostatic processes. It is well known that the inflammatory state differs in males and females, whether in animal models or in humans, with females having a more moderate response to inflammatory stimuli and a faster resolution of inflammation compared to males. This dissimilar response could determine a different efficiency of the cardiac repair mechanisms in the two sexes [3].

Deciphering the pathophysiological mechanisms underlying cardiac repair is, therefore, critical to favorably affect cardiac wound repair and to prevent development of HF. In this review, we will summarize the role of β-adrenergic receptors and estrogen in regulating the inflammatory and reparative response of the infarcted heart.

## 2. Cardiac Repair after Myocardial Infarction

**Figure 1:**
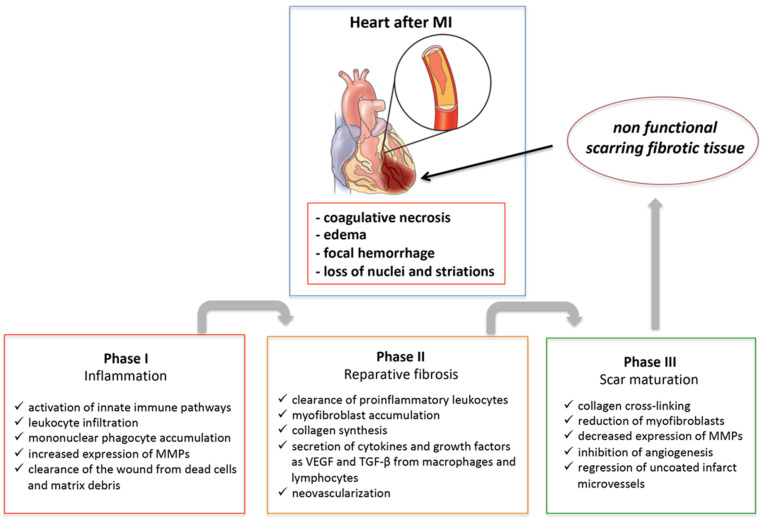
Schematic representation of the healing process of the myocardium after acute myocardial infarction (MI). At the end of the process, the result will be the structural but not functional restoration of the heart. MMPs, matrix metalloproteinases; VEGF, vascular endothelial growth factor; TGF-β, transforming growth factor-β.

Healing of MI is a dynamic process that can be schematically divided into three phases: inflammation following necrosis, reparative fibrosis, and scar maturation (Figure 1). When ischemia is of sufficient duration, the myocardium undergoes coagulative necrosis with edema, focal hemorrhage, loss of nuclei and striations, and granulocyte infiltration in the first days following myocardial ischemia. Consequentially, mononuclear phagocytes accumulate in the infarcted area with the task of clearing it of dead cells and matrix debris [4]. Inflammatory cells and necrotic myocytes secrete and activate matrix metalloproteinases (MMPs), which degrade the matrix, helping macrophages in resorption of necrotic tissue. During a later phase (days 3 to 7), inflammation attenuates and a reparative phase follows. This phase is characterized by the stimulation of collagen synthesis due to the conversion of fibroblasts into myofibroblasts, production of vascular endothelial growth factor (VEGF) and TGF-β1, neovascularization of the granulation tissue, and scar formation. Eventually, the scar will mature through increased collagen cross-linking, myofibroblasts will undergo apoptosis and be removed, and the inflammatory response will resolve. Therefore, timely suppression and resolution of the post-MI inflammation are required to ensure optimal repair and to prevent alterations in chamber shape and ventricular function. Excessive early inflammation may enhance matrix degradation, favoring cardiac rupture and formation of ventricular aneurysms. On the other hand, a prolonged inflammatory response may impair collagen deposition during the reparative phase leading to the formation of a weaker scar, induce an additional loss of CMs in the surrounding non-infarcted myocardium, and provoke a ventricular chamber dilatation.

CFs represent the sentinels of the heart as they perceive chemical, mechanical, and electrical stress signals and trigger the correct responses in case of cell or tissue damage [5,6]. Through a phenotypic switch to myofibroblasts, they allow remodeling and repair of the damaged area. In fact, they are able to secrete proteins of the extracellular matrix (ECM) and to express contractile proteins [7]. Furthermore, the proliferation and migration of CFs guarantee cardiac structural continuity in the event of CM loss through the formation of scarring fibrotic tissue [8].

## 3. β-Adrenergic Receptors

β-adrenergic receptors (β-ARs) regulate cardiac function in both physiological and pathological conditions [9]. It has been observed that they regulate inflammatory processes and, when activated in case of damage, especially β2-AR acts to maintain contractility and cardiac output [10,11].

β-ARs control a plethora of physiological functions by transducing signals mediated by catecholamines and neurotransmitters from outside the cell through the activation of intracellular signaling pathways. Similar to other G-protein-coupled receptors (GPCRs), β-ARs have a seven-stranded transmembrane quaternary conformation and are capable of signaling via G-protein and arrestin-dependent pathways [12]. In contrast to α-ARs, β-ARs respond powerfully to isoproterenol, considered a nonselective agonist for β-ARs, and to the physiological ligands adrenaline and noradrenaline. Three subtypes of β-ARs with different functions and tissue distribution have been identified. In humans, β1-AR is highly expressed in the heart, β2-AR is almost expressed in all tissues, and β3-AR is expressed mainly in the ovary, gallbladder, and smooth muscle [13]. Although in some circumstances β-ARs can pair to Gi protein, which inhibits cAMP production, all three β-ARs primarily couple to Gs protein by activating adenylyl cyclase. The increase in cAMP activates PKA, which induces a transient intracellular increase of Ca^2+^, which, in turn, stimulates further release of calcium from the sarcoplasmic reticulum (SR). This calcium-induced release of calcium results in the activation of troponin C and consequent contraction of cardiomyocytes. On the other hand, PKA, through phospholamban phosphorylation, allows calcium reabsorption within the SR while also regulating the cardiac relaxation phase [14].

Furthermore, it has been reported that β2-AR and β3-AR can also transduce the signal through mechanisms independent of the G protein [15].

The analysis of the structure of β-ARs complexed with agonists, inverse agonists, and antagonists has allowed us to obtain valuable information on the selectivity of the various ligands, providing models for drug design and important information on the mechanism of activation and signaling of these receptors (Table 1 lists the main β-AR agonists and antagonists). β-AR ligands have been classified as either agonist (full or partial) or antagonist with respect to G protein-coupling efficiency [16]. Recent research has expanded this classification to include the concept of inverse or biased agonism. The inverse agonists decrease constitutive cAMP accumulation, and the biased agonists, such as carvedilol, are antagonists for G protein-mediated signaling while simultaneously being agonists for G protein-independent signaling.

Due to their presence in the central nervous system and in effector organs such as the heart, vascular system, and adipose tissue, β-ARs mediate important homeostatic responses.

## 4. β-Adrenergic Signaling in the Post-Infarction Inflammatory Response

Inflammatory cells, including granulocytes, monocytes, and lymphocytes, broadly express β-ARs that mediate cell responses to endogenous catecholamines, norepinephrine, and epinephrine released from the adrenal medulla and sympathetic nerve termini. Among the three known β-ARs, subtypes β1- and β2- are able to modulate both innate and adaptive immunity and to influence cytokine production. The release of catecholamines after sympathetic nervous activation caused by the stress, for example, can affect the inflammatory response. Indeed, it has been reported that both people subject to chronic social stress (low socioeconomic status) and mice subject to repeated social defeat show upregulation of a subpopulation of immature pro-inflammatory monocytes (Ly-6c (high) in mice and CD16(-) in humans) within the circulating leukocyte pool in peripheral blood mononuclear cells [17]. In mice, this effect is ascribed to increased myelopoietic output of Ly-6c (high) monocytes and is inhibited by treatment with β-AR antagonists [17]. These results suggest that elevation of plasma catecholamine levels, induced by pain, anxiety, and/or a fall in cardiac output or arterial blood pressure during MI, can mobilize leukocyte progenitor cells from the bone marrow via β-AR stimulation and amplify the inflammatory response in the infarcted area. Also, stress-induced catecholamines can alter neutrophil trafficking [18,19] and promote formation of neutrophil–platelet co-aggregates [20]. However, it has also been found that the selective stimulation of the β-AR subtypes can have opposite effects. Indeed, activation of β2-AR, which is the most expressed β-AR isoform in immune cells in both rodents and humans [21], leads to anti-inflammatory actions by regulating cytokine production from leukocytes, migration of neutrophils and monocytes, as well as cell adhesion to activated endothelial cells [22,23,24,25]. Moreover, both immune cell activity and leukocyte migration to the heart following acute MI are impaired in the absence of β2-ARs [10]. In this setting, there is splenic retention of leukocytes due to the increased expression of vascular cell adhesion molecule 1 (VCAM-1) and the altered recruitment and trafficking of leukocytes to the injured heart, leading to impaired scar formation, followed by rupture and death. Recruitment and trafficking of leukocytes to the heart occur through the action of chemokines on leukocyte receptors to promote their migration to the site of injury [26]. Specifically, β2-AR is critical in regulating chemokine receptor 2 (CCR2) expression and leukocyte recruitment to the injured heart, as well as responsiveness to CCL2 chemokine ligand. This occurs via a β-arrestin2–biased signaling pathway involving activator protein 1 (AP-1), thus, suggesting that β-AR antagonists can affect cardiac repair. Indeed, it has been found that chronic β-blocker treatment with ICI 118,551 or carvedilol increases splenic leukocyte accumulation and decreases migration of leukocytes to the heart following acute MI, increasing cardiac rupture events [27]. To the contrary, activation of the β1-AR subtype is associated with pro-inflammatory responses in monocytes [28]. Specifically, β1-AR activation in monocytes concurrently stimulated with lipopolysaccharide increases IL-1β secretion through a protein-kinase A (PKA)-dependent mechanism. Furthermore, metoprolol, a β1-blocker, significantly inhibits neutrophil inflammatory responses both in patients and in animal models of cardiac ischemia, resulting in less severe reperfusion injury and smaller infarcts. This effect is abolished in neutrophil-depleted mice and when neutrophils are prevented from interacting with platelets. Additionally, the beneficial effects of metoprolol are also abolished by genetic ablation of β1-AR and rescued by restitution of β1-AR expression in hematopoietic cells [29].

Other evidence of a role for β-ARs in the pathogenesis of rupture and infarct expansion comes from preclinical experiments in which the β-ARs are over-expressed. Transgenic mice overexpressing the β2-AR had a lower risk of cardiac rupture during the acute phase after infarction despite the markedly enhanced left ventricle (LV) contractility and heart rate. Indeed, a significantly lower incidence of LV rupture was observed in transgenic than in wild-type mice 3–5 days after MI, despite a similar infarct size between the two groups [30]. Morphologic analysis showed a more severe infarct expansion in wild type versus transgenic mice. Additionally, collagen content was higher in both non-infarcted and infarcted areas of transgenic mice than in wild-type mice. Collectively, these results suggest that in leukocytes, the cardiac inflammatory response after MI could be regulated by modulating the β-AR signaling. In Table 2, the role of β1-ARs and β2-ARs in cardiac repair following MI in preclinical studies is reported.

## 5. β-Adrenergic Signaling in the Post-Infarction Reparative Phase

CFs are one of the most abundant cell populations in the heart, accounting for approximately 20–70% of cardiac cells, depending on the species, and have a key role during the post-MI reparative phase by regulating the amount of collagen as well as the production of cytokines, growth factors, and MMPs [33]. CFs mainly express the β2-AR subtype [34]. Proliferation and increased cytokine production, especially IL-6, follow chronic stimulation of β-ARs [35], and these effects are inhibited by treatment with non-selective or β2-AR selective antagonists but not with selective antagonists for β1-ARs. Additionally, treatment with isoproterenol and norepinephrine (non-selective β-AR agonists), as well as salbutamol (selective β2-AR agonist), triggers autophagy of cardiac fibroblasts, and this effect is inhibited by propranolol (non-selective β-AR antagonist) and ICI-118,551 (selective β2-AR antagonist) but not by atenolol (selective β1-AR antagonist) [33]. Noradrenaline, isoproterenol, and clenbuterol (a selective β2-AR agonist) all induce the proliferation of cardiac fibroblasts from multiple species (human, rat, rabbit) [36]. These mitogenic effects of noradrenaline and isoproterenol are inhibited by non-selective β-antagonists or β2-selective antagonists but not by β1-selective antagonists or α-AR antagonists. The use of pharmacological inhibitors and genetic manipulations of β-AR signaling indicates that fibroblast proliferation occurs through Gαs/ERK1/2-dependent IL-6 production and that β2-AR activation opposes the expansion of the infarct area [11]. The beneficial effects of β-blockers on adverse myocardial remodeling are likely to be due to a combination of effects on CM and CF function, the latter being due specifically to the β2-AR blockade.

In response to hormones, growth factors, and pro-inflammatory cytokines, the levels of which are increased during MI, CFs differentiate in myofibroblasts and produce pro-fibrotic molecules such as collagen and fibronectin, activating the system of MMPs and their inhibitors. Myofibroblasts, which are not observed in the normal myocardium, become highly proliferative and invasive and actively remodel the cardiac interstitium by increasing secretion of ECM-degrading MMPs and increasing collagen turnover, synthesis, and deposition [37,38,39]. These changes are important to ensure the repair of post-ischemic heart damage and the structural integrity of the heart after loss of CMs, thus, avoiding the progressive ventricular aneurysmal dilation. Myofibroblast density is markedly reduced in the mature scar, which is mainly characterized by cross-linked collagen and progressive loss of the cellular elements. The effects of β2-AR activation on ECM turnover, myofibroblast differentiation, and secretion of cytokines and growth factors at this stage of cardiac repair are less clear. Treatment with the β-blocker practolol reduces subepicardial scar formation and the percentage of total myocardium involved with the scar in an animal model of MI. Thinning of the left ventricular wall at the lateral margin of scar region suggests that β-AR blockade, during the post-MI repair process, can result in a reduced quantity and altered distribution of mature scar [31]. Cardiac cavity enlargement and scar thinning were also found in rats with MI after long-term treatment with the β-blocker propranolol, suggesting that β-blockade may affect scar amount [32].

## 6. Estrogen and Estrogen Receptors

It has been hypothesized that estrogen, particularly 17β-estradiol (E2), may facilitate cardiac repair and remodeling processes, although the exact mechanisms have not yet been identified.

In addition to female gonads, E2 can be produced locally in other tissues as a product of the enzymatic conversion of testosterone by the aromatase, an enzyme expressed in different tissues such as fat, bone, brain, heart, and blood vessels of both the sexes [40]. This increases the potential importance of estrogen in cardiovascular physiology.

E2 exerts its actions through the interaction with specific estrogen receptors ERα (three isoforms: ERα66, ERα46, and ERα36) and ERβ present in the cytosol and nucleus, in the intracellular compartments such as the endoplasmic reticulum, Golgi and mitochondria, and on the plasma membrane [41]. Another estrogen receptor, called the G-protein-coupled estrogen receptor (GPER), has been found on the plasma membrane and in several intracellular organelles [42]. Interestingly, GPR30 shares structural features and biochemical pathways with βARs, and this could be the basis of their interaction and functional overlap [43].

Both ERα and ERβ are present in human heart tissue, in particular, in the sarcolemma and intercalary discs of human cardiac muscle (ERα) and in ventricular and atrial cells (ERβ) [44,45,46]. However, conflicting evidence exists regarding ER expression and localization in CMs. Additionally, due to the dubious specificity of the antibodies [47], the expression data of the various estrogen receptor isoforms are still contradictory. Recently, a study conducted at the mRNA level in rat cardiovascular tissues revealed high levels of expression of ERα, followed by GPER in terms of abundance, while ERβ appeared almost undetectable [48]. Estrogen affects the function of the cardiovascular system via genomic and non-genomic actions. Genomic action is mediated by ERα and ERβ. After interaction of E2 with its receptors, these latter change their conformation and migrate as homo- or hetero-dimers into the nucleus, where they regulate the transcription of a number of genes. Alternatively, the regulation of transcription can occur indirectly through the mediation of cofactors (e.g., SP-1, AP-1, or NF-κB) that bind DNA [49]. Despite their predominant cytoplasmic localization and genomic effects, ERs can also promote non-genomic cell signaling when embedded in the cell membrane. When estrogens bind to membrane receptors, rapid signaling pathways are activated, generally consisting of phosphorylation of membrane-associated proteins.

For example, through this rapid signaling pathway that does not involve transcriptional activation, E2, through the binding with ERs, activates nitric oxide synthase (eNOS) in endothelial cells by stimulating phosphatidylinositol 3-kinase (PI3K) with consequent increase in NO production [50]. Through the activation of the PI3K/Akt pathway, E2 can also inhibit the apoptosis induced by some transcription factors [51]. As for GPER, after E2 binding, it rapidly activates membrane signaling pathways involving kinases, ion channels, and various second messengers [42]. It has also been suggested that GPER could indirectly regulate gene expression through an importin-dependent mechanism [52].

## 7. Estrogen in Cardiac Repair

Studies of the effects of estrogen on cardiac repair following acute MI have revealed conflicting results. Female C57BL/6J mice show a decreased rate of cardiac rupture during the acute phase of MI [53]. Moreover, ovariectomy in C57BL/6J mice yields a reduced infarct size both one day (18% decrease; *p* < 0.01) and six weeks (14% decrease; *p* < 0.05) after MI. However, despite having smaller infarcts, E2 replacement also increases left ventricular mass and mortality in the infarcted ovariectomized (OVX) animals [54].

**Figure 2:**
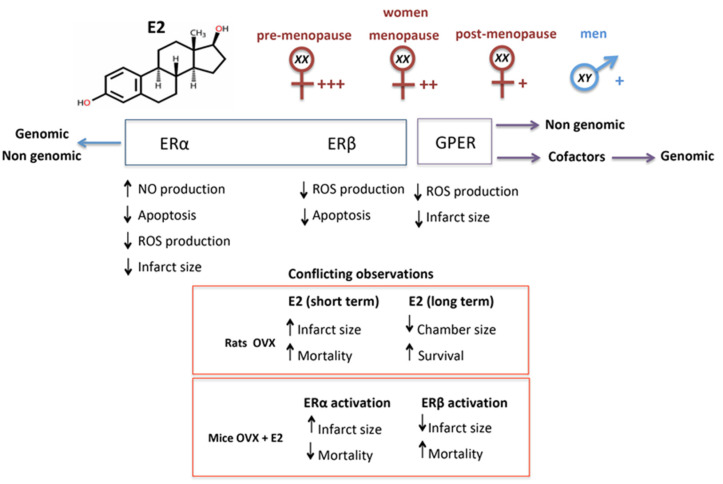
Schematic representation of the role played by estrogen in cardiac repair and of the effects mediated by specific receptors. E2 through its interaction with the different receptors (ERα, ERβ or GPER) can induce different effects, which can be genomic and/or non-genomic. Importantly, E2 levels vary by sex and, especially in the case of females, by age. Postmenopausal women may have E2 levels similar to those found in men. Furthermore, E2 can sometimes induce discordant effects based on the experimental models used. E2, estradiol; GPER, G protein-coupled estrogen receptor; OVX, ovariectomized; NO, nitric oxide; ROS, reactive oxygen species.

In OVX Wistar female rats, E2 replacement did not prevent the MI-induced changes in cardiac function, collagen deposition, and myocyte hypertrophy [55]. Furthermore, E2 treatment was associated with an increase of infarct size and a trend toward increased mortality at the time of MI or early post-MI period. However, long-term treatment with E2 normalizes ventricular wall tension and chamber dimension in MI survivors, promoting increased survival [56]. Furthermore, OVX rats subjected to ischemia-reperfusion (I/R) present significantly decreased post-ischemic recovery of left ventricular function and increased MI extension, and these deleterious effects were avoided by E2 replacement [57]. In OVX-MI mice, activation of ERα reduces mortality in spite of increasing infarction extension, while stimulation of ERβ accounts for reduced infarction area and increased cardiac hypertrophy and mortality [58]. E2 treatment also reduces cardiomyocyte apoptosis in the infarction zone in OVX-MI mice (Figure 2). The conflicting results reported by the literature may depend on the different experimental models considered.

## 8. Notes on the Role of Estrogen in Cardiac Regeneration

The clinical management of HF that occurs following MI has considerably improved, but to date, there are no therapeutic strategies capable of restoring the functionality of the infarcted myocardial areas. Although a cardiomyocyte turnover has been observed in the adult mammalian heart, including in humans [59], as terminally differentiated cells, adult CMs are unable to proliferate to replace those lost following the ischemic events.

The only possibility to restore cardiac function would, therefore, be the regeneration of cardiomyocytes starting from stem cells present in the myocardium or in other body districts and recruited into the damaged myocardium [60,61,62].

Some experimental studies carried out in animal models in recent years have highlighted not only the presence of endothelial progenitors (EPCs) in the bone marrow (BM) but also their mobilization towards the myocardium following damage. It has been shown that EPCs present in the infarcted myocardium, which come from the BM, are receptor tyrosine kinase protein (c-Kit)-positive and predominantly express ERα. Some studies suggest that these EPCs c-Kit^+^/ERα^+^ can improve cardiac function both in animals and humans in post-MI [63] due to their potential for self-renewal, proliferation, and differentiation following acute ischemic injury [64].

**Figure 3:**
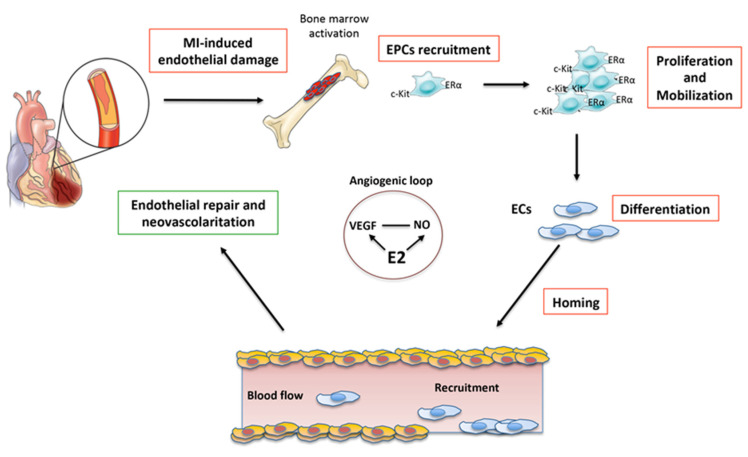
Schematic representation of the neovascularization following acute myocardial infarction (MI). E2 induces an angiogenic loop by stimulating VEGF and NO production. E2 also promotes proliferation, mobilization, homing, and differentiation of EPCs into ECs, thus, favoring the re-endothelialization and revascularization of the damaged myocardium. E2, estradiol; EPCs, endothelial progenitor cells; ECs, endothelial cells; NO, nitric oxide; VEGF, vascular endothelial growth factor; c-kit, type III receptor tyrosine kinase; ERα, estrogen receptor α. This cartoon was inspired by Nova-Lamperti et al., 2016 [74].

Although EPCs are potentially able to differentiate into CMs, at least in vitro, the beneficial effects observed in vivo appear to be essentially related to the neovascularization of the area affected by the infarction. The EPCs that participate in the normal turnover of the endothelium are massively mobilized by the bone marrow towards the myocardium in case of stressful events, such as acute MI, where they contribute to neovascularization [65] (Figure 3). This would suggest that by increasing the mobilization and/or homing of EPCs at the site of damage, cardiac repair could be enhanced. Thus, it is extremely important to know the molecular mechanisms underlying these processes. The analysis of the signaling pathways involved in cardiac regeneration highlighted the involvement of Hippo, MAPK, and PI3K-AKT pathways [66,67], which are also involved in various aspects of the tumor progression. This makes their exogenous modulation particularly tricky and potentially dangerous.

E2 has a wide range of effects on the heart, including the prevention of apoptosis and the reduction of damage induced by I/R [68]. Several studies have indicated that E2 is able to favor the neovascularization of ischemic tissues by favoring the recruitment of EPCs at the damaged site [60,69,70]. Furthermore, in accordance with the above, it was observed that E2 treatment in the acute phase of MI reduced cardiomyocyte apoptosis through activation of the PI3K/Akt signal [71]. It would seem that the protective effects of estrogen are related to the expression of ERα and ERβ both at the level of vascular endothelial cells and CMs. However, studies conducted on animal models KO for the different estrogen receptors have shown that the three receptors, ERα, Erβ, and GPR30, play a cardioprotective role with genomic and non-genomic mechanisms [72], although ERα would seem to have a predominant role [68,73].

## 9. Interplay between ERs and β-ARs and Its Potential Therapeutic Value

The mechanisms and biochemical pathways triggered by ERs (ERα, Erβ, and GPR30) and β-ARs (β1-AR, β2-AR, and β3-AR) are strongly intertwined and widely shared. The importance of this cross talk in the pathophysiological regulation of the cardiovascular system has been hypothesized since the early 2000s and subsequently demonstrated in numerous in vitro and in vivo models. Depending on the experimental conditions and the models used, the activation of ERs can sometimes synergize with βAR action and, at other times, oppose it [75,76]. These observations, together with the co-presence of ERs and β-ARs in cardiomyocytes and endothelial cells of both sexes, have suggested their possible implication in gender-specific cardiovascular regulation.

It is recognized that ERs co-regulate, along with β-ARs, intracellular calcium levels in cardiac cells [77]. Numerous studies have, in fact, shown that estrogens were able to increase calcium-regulated proteins [78,79], including ATPase and membrane channels in different cell types [80,81,82,83,84]. Therefore, ERs-mediated signaling can have profound implications in the regulation of cardiac contractile activity, physiologically under the control of ARs [85,86,87].

Furthermore, ERs directly modulated the expression level of β-ARs in different experimental conditions. In particular, in cardiac cells estrogen downregulated β1-AR and upregulated β2-AR expression [88].

As mentioned above, the similarity and the partial overlap of the evoked biochemical pathways were observed between GPR30 and β-ARs. Interestingly, estrogens, through the involvement of GPR30, were able to mitigate the negative effects of catecholamines in a model of stress-induced cardiomyopathy [89]. In particular, favoring the coupling of β2-AR to the Gαs-mediated signal while disfavoring that mediated by Gαi, estrogen induced a rapid increase of cAMP and PKA activity both in OVX female rats and in cultured CMs [88].

Additionally, GPR30 and β2-ARs, converging on the (PI3K)/Akt pathway, were able to inversely modulate the Bcl-2 and Bax proteins protecting cardiomyocytes from ischemia-induced apoptosis [90,91].

The localization of both β-ARs and ERs inside the caveolae has been observed to affect their function [92]. For instance, in vascular cells, the ability of the two receptors to cooperate through the eNOS signal pathway is bound to their localization within the caveolae [93,94]. However, the relevance of the interaction among ERs, caveolins, and β-ARs in the pathophysiology of cardiomyocytes is still debated [92]. The deepening of the knowledge of the cross talk between ERs and β-ARs appears, therefore, critical in the clinical management of cardiovascular diseases. In fact, β-blockers are still widely used, together with calcium channel blockers, in the treatment of cardiovascular diseases [95]. Importantly, it has been observed that the efficacy of both β- and Ca^2+^-blockers varies according to sex and age, precisely on the basis of the interaction between estrogen and β-ARs [88]. If, as the experimental data would seem to indicate, β-ARs expression and activity can be influenced by estrogen [88], men and women could respond differently, also in relation to age, to this class of drugs. Having this information could guide the physician towards a personalized therapy taking into account, among other things, the age and gender of the patients [91].

## 10. Conclusions

The therapeutic goal after acute MI would be mainly to reduce the size of the myocardial scar, which, while ensuring the structural integrity of the heart, is made up of non-functional tissue. This should minimize the probabilities of later developing HF.

At the moment, the best treatments for HF appear to be those that can reduce or reverse ventricular remodeling. For example, drugs such as β-blockers significantly improve either symptoms or survival of patients and are considered extremely important today in the therapy of HF [96]. Although the β-blockers are currently the standard treatment after MI and HF [97], in apparent contradiction to this, some papers show that β2-AR agonists are capable of reducing the size of the infarcted area [98,99]. In agreement with this, it was found that β2-AR signaling would have a positive effect on the proliferation and survival of EPCs [100]. Interestingly, a sex dimorphism has also been observed with regard to EPCs, strongly influenced by hormonal environment, in particular, by estrogen.

The differences between men and women in the outcome of numerous cardiovascular diseases, including MI, have been recognized for long time and largely related to gender and sex hormones. In the same vein, sex- and/or gender-related pharmacological differences have also been widely demonstrated. For example, the integration between biochemical pathways activated by sex hormones and those activated by β-ARs has been reported to prevent the upregulation of β1-AR in the heart [101,102,103]. Regarding β-blockers, a greater reduction in blood pressure and a higher incidence of adverse events in women has also been reported due to the higher plasma levels that are achieved in women [104]. Accordingly, the sex could also influence the expression and signaling of β-ARs in animals, where a higher density of β-ARs on male CMs compared to female CMs was observed [105].

The repair processes following MI appear very complex and regulated by a plethora of factors, including β-ARs and sex hormone receptors, especially ERs. In addition, the literature data often appear contradictory, perhaps due to the different experimental models used in the studies. Further research will be, therefore, needed to understand how repair mechanisms are driven and influenced by the interaction between adrenergic signaling and the hormonal environment before this crosstalk could play a role as a possibly gender-specific therapeutic target in the prevention of post-infarct HF.

## Figures and Tables

**Table 1:** List of selected β-adrenergic receptor agonists and antagonists.

β-AR Agonists	β-AR Antagonists
Adrenaline non-selective β-AR physiological ligand	Propranolol non-selective β1 and β2-AR
Noradrenaline non-selective β-AR physiological ligand	Metoprolol selective β1-AR
Isoprotere nolnon-selective β-AR	Bisoprolol selective β1-AR
Dobutamine selective β1-AR	Carvedilol non-selective β1 and β2-AR
Salbutamol selective β2-AR	Atenolol selective β1-AR
Clenbuterol selective β2-AR	Practolol selective β1-AR
Salmeterol selective β2-AR	Sotalol non-selective β1 and β2-AR

**Table 2:** β-ARs and cardiac repair following myocardial infarction in preclinical studies.

Receptor	Effects	Mechanisms	Refs.
**β2-AR**	Impaired scar formation followed by cardiac rupture and death in β2-AR KO mice subjected to MI	Decreased migration of leukocytes to the injured heart	[10]
**β2-AR**	Chronic β-blocker treatment with ICI 118,551 or carvedilol increases cardiac rupture events after MI	Splenic leukocyte accumulation and decreased migration of leukocytes to the injured heart	[27]
**β1-AR**	Treatment with metoprolol, a β1-blocker, reduces infarct size in animal models of cardiac ischemia	Inhibition of neutrophil-driven inflammatory responses	[29]
**β2-AR**	Transgenic mice overexpressing the β2-AR have lower incidence of cardiac rupture following MI	Increased production of collagen	[30]
**β2-AR**	β2-AR activation opposes the expansion of infarct area	Increased production of collagen and fibroblast proliferation	[11]
**β-ARs**	Chronic treatment with nonselective β-blockers causes cardiac cavity enlargement and scar thinning	Reduced scar amount	[31,32]
